# Nature of the Syphiltic Virus

**Published:** 1856-04

**Authors:** 


*Nature of the Syphiltic Virus.*

M. CASTANO, Physician-Major in the army of the East, regards syphilis as resulting from a vegetable fungiform parasite, the germ of which is developed in the tissues. The cure of venereal affections therefore consists in destroying this new body and the climination of its spores and sporules from the system. The metallic anti-syphilities destroy the parasite and render the spores incapable of germination.—*Medical Counselor.*

